# Two new species of *Bryobia* (Acarina, Prostigmata, Tetranychidae) from South France

**DOI:** 10.3897/zookeys.480.9166

**Published:** 2015-02-02

**Authors:** Philippe Auger, Tea Arabuli, Alain Migeon

**Affiliations:** 1 INRA, UMR 1062 CBGP (INRA, IRD, CIRAD, Montpellier SupAgro), Campus de Baillarguet, 34988 Montferrier-sur-Lez, France; 2 Institute of Entomology, Agricultural University of Georgia. University Campus at Digomi David Aghmashenebeli Alley, 13-th km, 0159, Tbilisi, Georgia

**Keywords:** Acari, Tetranychidae, new species, Leguminosae, France

## Abstract

Two new species of Tetranychidae belonging to the genus *Bryobia* are reported from France. *Bryobia belliloci* **sp. n.** and *Bryobia gigas* **sp. n.** collected on *Genista cinerea* and *Bituminaria bituminosa*, respectively, are described and illustrated in the present work. Additional data to the original description of *Bryobia cinereae* are given and an identification key to known *Bryobia* species from France is also provided.

## Introduction

Among forty eight species of Tetranychidae recorded from France fourteen species belong to the genus *Bryobia* Koch, 1836, six of them being endemic to this country (Migeon and Dorkeld 2006–2014). Recent sampling efforts conducted near the Mediterranean coast disclosed two new species of tetranychid mites belonging to this genus. In the current paper we report their descriptions, we complete the description of *Bryobia cinereae* with measures and drawings of characters usually not reported in descriptions and we provide a key to the fourteen species of *Bryobia* known from France.

## Material and methods

Mites were collected directly from field samples in 70% ethyl alcohol. Following clearing in lactic acid (50%) for 24 hours they were mounted in Hoyer’s medium. The specimens were examined using a Leica DM LB 2 phase contrast microscope and illustrated with the aid of a drawing tube attachment (*camera lucida*). Measurements were taken using the imaging software Perfect Image® (Clara Vision) coupled with ProgRes® Capture Pro 2.6 software for image acquisition. The setal nomenclature used in the descriptions follows Lindquist (1985). Leg setal counts are given in the order: coxa-trochanter-femur-genu-tibia-tarsus. Numbers of setae refer to tactile setae, solenidia are given in parentheses and alternative counts are given in brackets. All measurements are given in micrometers and correspond to the holotype followed (in parentheses) by minimum and maximum values from paratypes. Setae were measured from the centre of their setal bases to their tips. The distance between two setae was measured as the distance from the centre of one setal base to the other.

## Systematics

### Family Tetranychidae Donnadieu, 1875 Subfamily Bryobiinae Berlese, 1913 Tribe Bryobiini Reck, 1952 Genus *Bryobia* Koch, 1836

#### Bryobia belliloci sp. n.

Taxon classificationAnimaliaProstigmataTetranychidae

http://zoobank.org/4C94F5E0-E6FC-4FDA-9BCD-30629D4703A6

**Figure 1.**
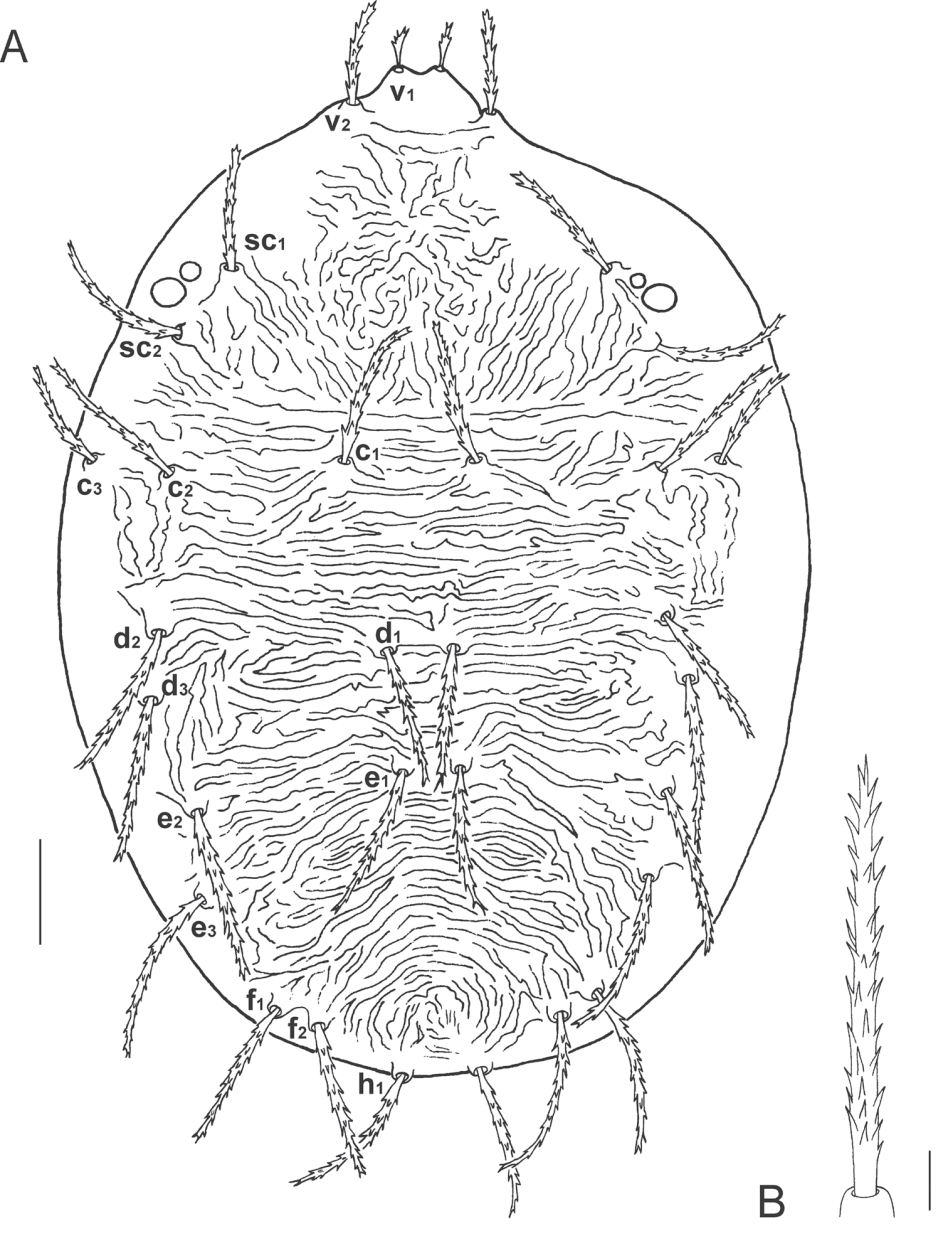
*Bryobia belliloci* sp. n., female: **A** dorsal aspect **B** dorsal *h*_1_ seta. Scale bars = 50 µm (**A**), 10 µm (**B**).

**Figure 2.**
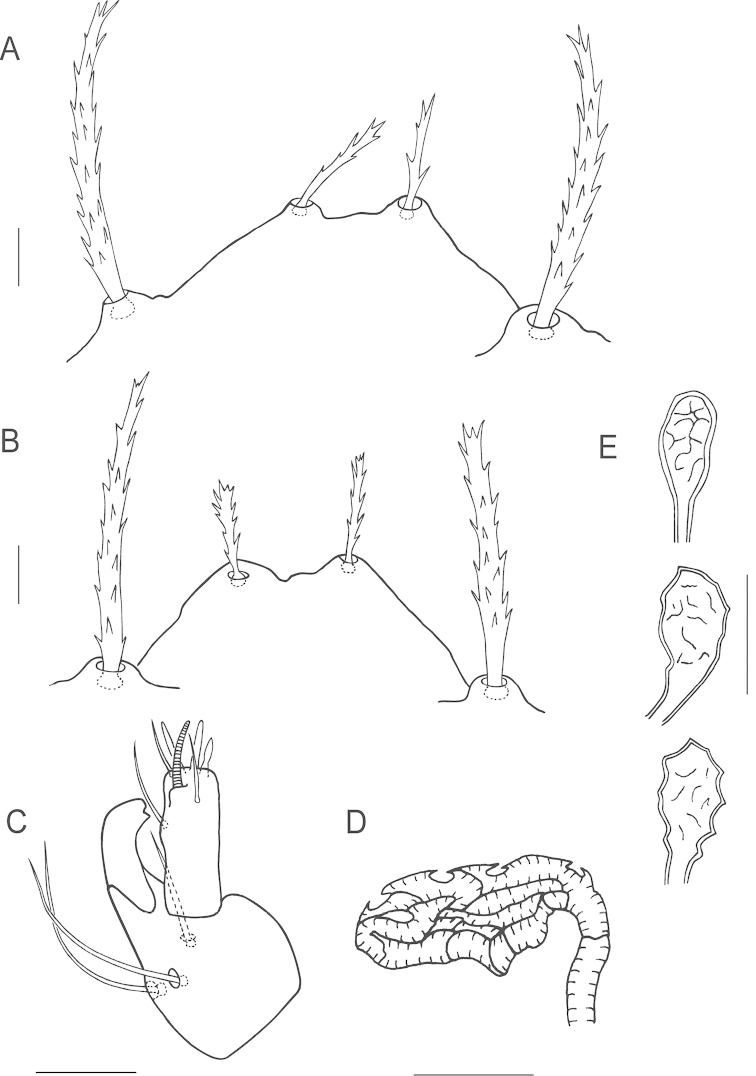
*Bryobia belliloci* sp. n., female: **A–B** prodorsal lobes (variations) **C** palpal tibia and tarsus **D** peritremal distal anastomosis **E** spermatheca (variations). Scale bars = 10 µm.

**Figure 3.**
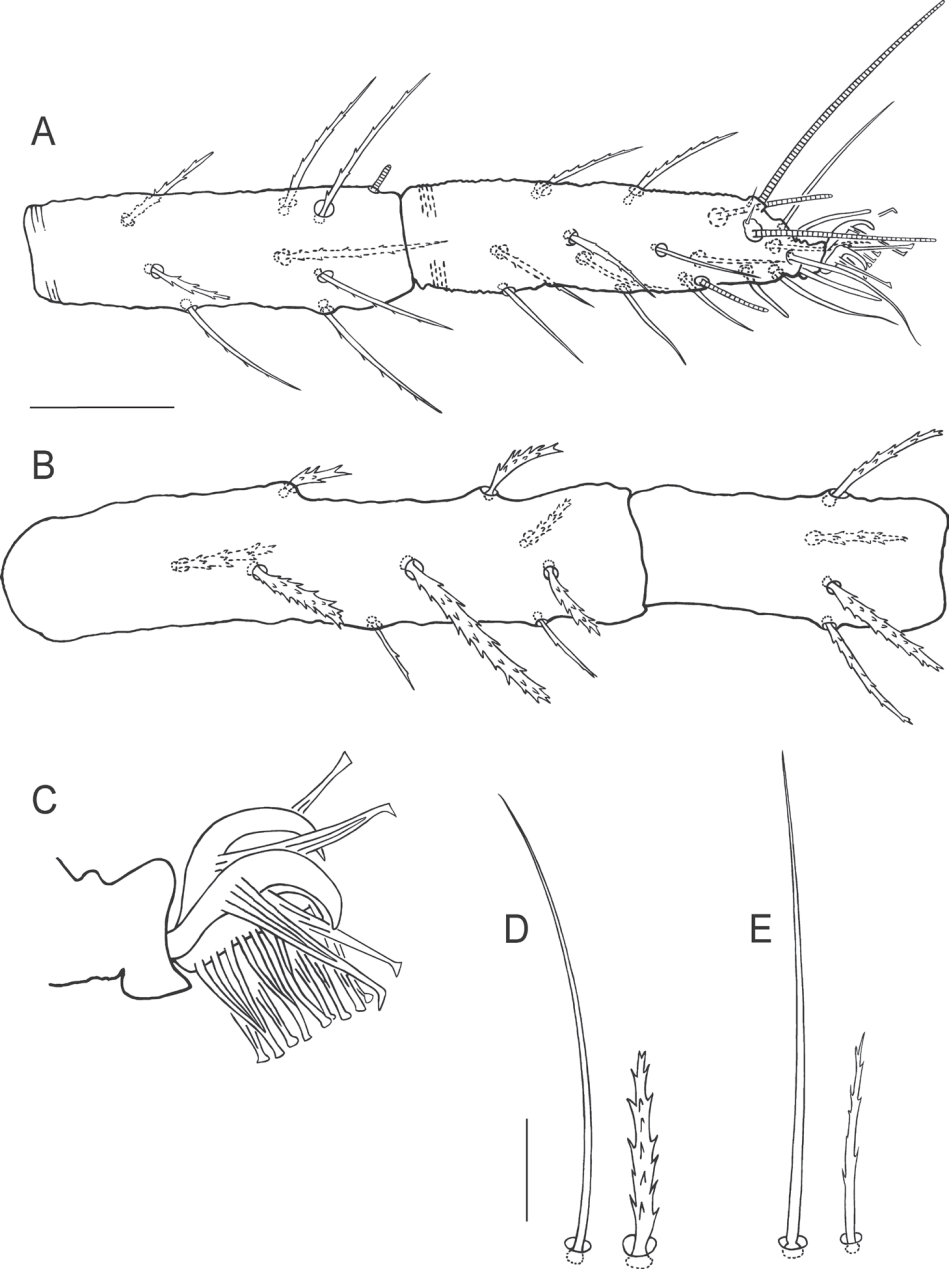
*Bryobia belliloci* sp. n., female: **A** tarsus and tibia I **B** genu and femur I **C** claws and empodia I–IV **D** coxisternal setae *1b* and *1c* **E** *Bryobia cinereae* Auger & Migeon (2014), Holotype female, coxisternal setae *1b* and *1c*. Scale bars = 25 µm (**A–B**), 10 µm (**C–E**).

**Figure 4.**
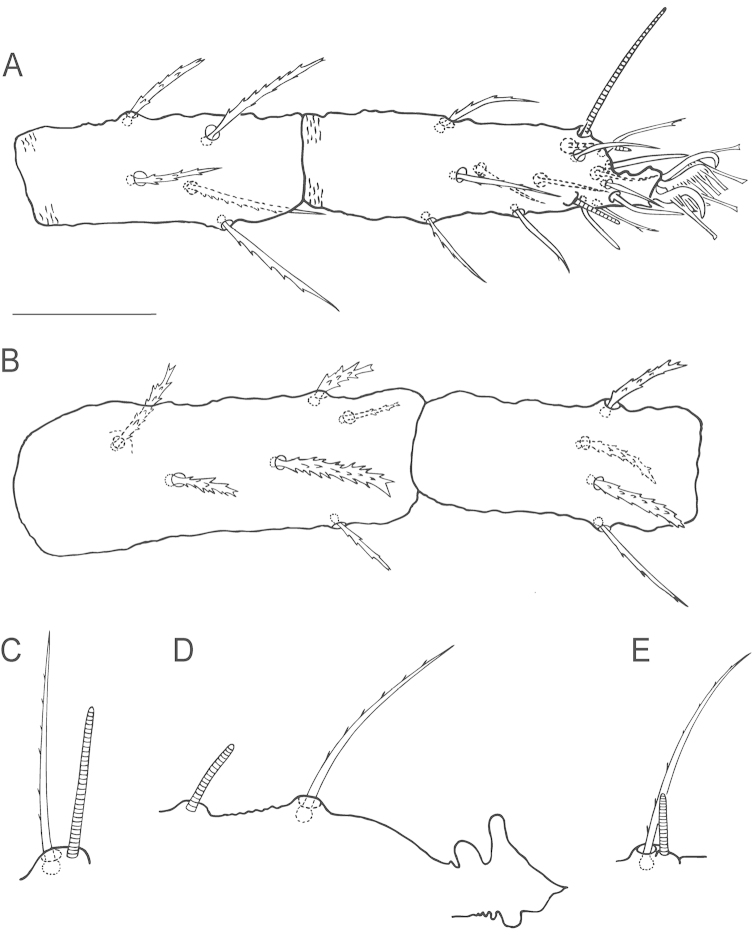
*Bryobia belliloci* sp. n., female: **A** tarsus and tibia II **B** genu and femur II **C** duplex setae on tarsus III **D** solenidion and associated tactile seta on tarsus IV **E** abnormal duplex setae on one tarsus IV of Holotype. Scale bars = 25 µm (**A–B**), 10 µm (**C–E**).

**Figure 5.**
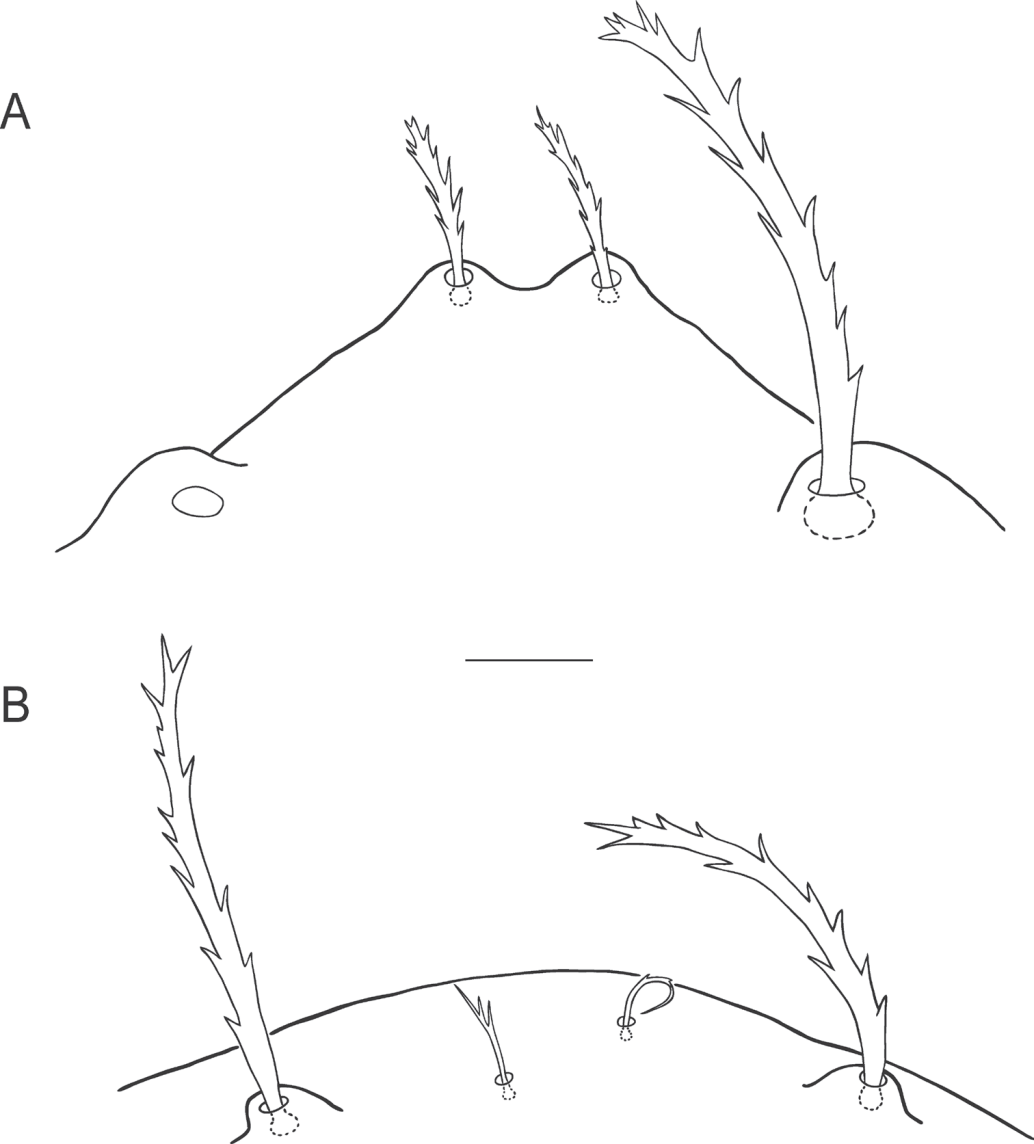
*Bryobia belliloci* sp. n., prodorsal anterior part: **A** deutonymphal prodorsal lobes **B** protonymphal vertical setae (*v*_1_, *v*_2_), absence of prodorsal lobes. Scale bar = 10 µm.

Figures 1 , 2 , 3 , 4 , 5

##### Type material.

Holotype (female), 22 female, 1 deutonymph and 1 protonymph paratypes on 25 microscopic preparations from *Genista cinerea* (Vill.) DC. (Leguminosae), Pla d’Auçà (42°35.28’ N, 2°20.58’ E, alt. 1,210 m), Serdinya, Pyrénées-Orientales (66), France, 6.VI.2013, leg. P. Auger and A. Migeon. All the material housed in the collection of the Centre de Biologie et de Gestion des Populations (CBGP), coll. Auger-Migeon N°1839 for holotype and 1840-1863 for paratypes, 34988 Montferrier-sur-Lez, France.

##### Diagnosis.

Limited anterior dorsal propodosomal projections over gnathosoma, outer prodorsal lobes scarcely developed more resembling to tubercle-like structures, inner lobes more developed, base almost fully fused, more or less cone-shaped, with wide shallow incision between inner vertical setae (*v*_1_). Dorsal setae elongate, serrate, with sharp tips, inserted on tubercles, subequal in length on hysterosoma, second pair of dorsocentral hysterosomal setae (*d*_1_) longer than distance to consecutive setae (*e*_1_) insertions, *f*_1_ and *f*_2_ setae marginal and contiguous. Empodia with two rows of tenent hairs.

##### Description.

FEMALE. Holotype 485 long (excluding gnathosoma) gnathosoma 110 long (measured to the tip of palps), width 352. 9 paratypes measured, 481–528, gnathosoma 104–120 long, width 318–354.

*Dorsum*. Prodorsum with four pairs of setae, with weakly developed anterior lobes (Figs 1A, 2A–B). Outer propodosomal lobes small, about 10 µm, more or less similar in length to dorsal tubercles; inner lobes with large fused base forming cone-shaped projection, incision between median lobes wide and shallow. Basal width of propodosomal lobes about 76 (74–82), distance between first (*v*_1_) and second (*v*_2_) pair of propodosomal setae insertions 19 (17–19) and 65 (49–70), respectively. *v*_2_ setae about 2.5 the size of *v*_1_. An imaginary transversal line joining tip of *v*_1_ setae crosses *v*_2_ setae about their two-thirds. Dorsal body setae elongated, slender, serrate, acute distally, inserted on tubercles, subequal in length (*v*_1,_ *v*_2,_ *c*_3_ setae smaller, *v*_1_ the smallest) (Fig. 1B). Dorsocentral setae *c*_1_ and *e*_1_ shorter than distances between consecutive setae, *d*_1_ longer than distance between setal insertions *d*_1_ and *e*_1_ (length of holotype and variations of 9 paratypes): *v*_1_ 20 (18–23); *v*_2_ 52 (48–54); *sc*_1_ 65 (65–71); *sc*_2_ 70 (62–70); *c*_1_ 74 (74–88); *c*_2_ 75 (66–75); *c*_3_ 55 (52–60); *d*_1_ 74 (71–81); *d*_2_ 80 (77–90); *d*_3_ 82 (78–91); *e*_1_ 79 (76–90); *e*_2_ 83 (79–91); *e*_3_ 83 (78–92); *f*_1_ 81 (76–85); *f*_2_ 84 (76–84); *h*_1_ 72 (69–73). Distances between setae: *c*_1_-*c*_1_ 69 (58–70), *d*_1_-*d*_1_ 33 (30–43), *e*_1_-*e*_1_ 26 (20–28), *c*_1_-*d*_1_ 91 (90–96), *d*_1_-*e*_1_ 59 (59–74). Sacral setae (*f*_1_ and *f*_2_) in marginal position and contiguous. Dorsal body surface wrinkled, on propodosoma irregular medially and mostly oblique laterally, transverse on hysterosoma, more or less arched in the distal part comprised between *e*_3_ and *h*_1_ setae. Area immediately anterior to *h*_1_ setae with fine arched reticulation.

*Gnathosoma*. Stylophore rounded, slightly emarginate anteriorly, longer than wide. Tibial claw of palpus bidentate (Fig. 2C). Palptarsus slightly elongated, longer than tibial claw, about 21 (19–21) long (including setae) with six setae and one solenidion. Eupathidia *ul*’ζ, *ul*’’ζ shorter than solenidion, *su*ζ shorter. Peritreme anastomosed distally in an oval enlargement: length 20 (20–26), width 9 (9–11) (Fig. 2D).

*Venter*. Striation transverse between 1^st^ (*1a*) and 2^nd^ (*3a*) pairs of setae, irregularly longitudinal (broken medially, oblique laterally) between 2^nd^ and 3^rd^ (*4a*) pairs of setae, transverse between members of *4a* and between *4a* and aggenital (*ag*) pairs of setae. Area immediately anterior to genital flap with irregular longitudinal striation, V-shaped between *ag* setae. Sacculus of spermatheca small and oval (Fig. 2E). Three pairs of pseudanal setae (*ps*_1–3_) and two pairs of ventrocaudal (*h*_2–3_) setae present.

*Legs*. Shorter than body length. Leg I 330 (317–335) long (measured from trochanter to tarsus), leg II 249 (237–250), leg III 245 (237–260), leg IV 272 (266–281). Length of segments of leg I as follows: trochanter 24 (21–30), femur 101 (92–108), genu 57 (50–57), tibia 71 (66–74), tarsus 78 (74–78). Leg setal counts as follows (Figs 3A–B, 4A–B):

I 2 − 1 − 9 [7-10] − 4 − 9 [8] + (1) – 17[16] + (2) + 2 duplexes;

II 1 − 1 – 6[5-7] − 4 –5[3-4] − 15[14] + (2) + 1 duplex;

III 1 − 1 – 4[3-5] – 2 [1-3] − 4[5] − 12[11] + 1 duplex;

IV 1 − 1 − 4 [3-5] – 2[3] – 6 [4-5] − 13[12-14] + (1).

True claws uncinate, with one pair of tenent hairs, empodial pads I-IV similar with two rows of ventrally directed tenent hairs (Fig. 3C). Internal lateral seta *l*’_1_ on femur I large 37 (34–40), serrated. Proximal coxisternal seta *1b* slender 45 (41–50), distal coxisternal seta *1c* shorter 18 (18–21), serrate, stout (Fig. 3D). Tarsus III associated setae serrate and approximate with solenidion forming duplex, tactile member longer and proximal, solenidion about ¾ the length of tactile (Fig. 4C); tarsus IV with solenidion well-separated from tactile, proximal, about 1/3 the length of tactile (Fig. 4D–E).

MALE: Unknown

DEUTONYMPH: one specimen measured, 520 long (including gnathosoma), width 330.

*Dorsum*. Prodorsal lobes similar in shape to females (Fig. 5A), prodorsal setae *v*_1_ and *v*_2_ elongated and serrate, *v*_2_ the largest about 3 times the length of *v*_1_; an imaginary transverse line joining the tips of *v*_2_ setae passes well the tips of *v*_1_. Dorsal body setae slender, needle-like and serrate, inserted on tubercles. Setae *c*_1_ and *e*_1_ shorter than distances between consecutive setae, *d*_1_ longer than distance between setal insertions *d*_1_ and *e*_1_. Setae *f*_1_ and *f*_2_ in marginal position, contiguous. Lengths of dorsal setae: *v*_1_ 15; *v*_2_ 44; *sc*_1_ 58; *sc*_2_ -; *c*_1_ 64; *c*_2_ 64; *c*_3_ 39; *d*_1_ 76; *d*_2_ 71; *d*_3_ 70; *e*_1_ 68; *e*_2_ 70; *e*_3_ 74; *f*_1_ 80; *f*_2_ 64; *h*_1_ 63.

*Legs*. Shorter than body length, leg I 233 long (including coxa). Internal lateral seta (*l*’) on femur I large. Leg setal counts as follows:

I 2 − 1 − 6 [5] − 4 − 5 + (1) – 13 + (1) + 2 duplexes;

II 1 − 1 – 3 – 4 – 3 − 11 + 1 duplex;

III 1 − 1 − 2 − 1 – 3 – 10 + (1);

IV 1 − 0 − 2 − 1 – 3 − 10.

True claws uncinate with one pair of tenent hairs, empodia with two rows of tenent hairs. Tarsus III with solenidion well-separated from tactile, proximal.

PROTONYMPH: one specimen measured, 355 long (including gnathosoma), width 240.

*Dorsum*. Prodorsal lobes absent, *v*_1_ very short, poorly indented, not inserted on tubercle, *v*_2_ larger, elongate and serrate, inserted on tubercle (Fig. 5B). Other dorsal body setae slender, serrate, inserted on tubercles except *c*_3_. Setae *f*_1_ and *f*_2_ in marginal position, not contiguous. Lengths of dorsal setae: *v*_1_ 8; *v*_2_ 37; *sc*_1_ -; *sc*_2_ 30; *c*_1_ -; *c*_2_ -; *c*_3_ 21; *d*_1_ -; *d*_2_ 46; *d*_3_ -; *e*_1_ -; *e*_2_ -; *e*_3_ 44; *f*_1_ 56; *f*_2_ 43; *h*_1_ 45.

*Legs*. Shorter than body length, leg I 196 long (including coxa). Internal dorsal row on femur I with one long seta finely serrate. Leg setal counts as follows:

I 2 − 0 – 3 − 4 – 5 + (1) – 9 + 2 duplexes;

II 1 − 0 – 3 − 4 – 3 − 9 + 1 duplex;

III 1 − 0 – 2 – 1 − 3 − 8;

IV 0 − 0 − 2 – 1 – 3 − 6.

True claws uncinate with one pair of tenent hairs, empodia with two rows of tenent hairs.

##### Remarks.

The combination of prodorsal lobes poorly developed, dorsal setae not spatulate but slender and leg setal counts brings this species very close to *Bryobia cinereae* Auger & Migeon, 2014. It can be distinguished from this species by the shape and the size of dorsohysterosomal setae: they are wider, stouter in *Bryobia cinereae* but longer in *Bryobia belliloci*. Thus, dorsocentral setae are shorter than the distance between consecutive setae in *Bryobia cinereae* whereas *d*_1_ setae surpass well *e*_1_ setal insertions in *Bryobia belliloci*. Noticeable differences are also present regarding the following morphological characters in *Bryobia belliloci*: 1) the incision between the inner prodorsal lobes is wider and shallower; 2) the four legs are longer; 3) the peritremal distal enlargement is shorter; 4) the internal lateral seta *l*’_1_ on femur I is longer (Table 1); 5) coxisternal setae *1b* and *1c* are similar in length compared to those of *Bryobia cinereae* (Table 1) but the coxisternal seta *1b* is serrate, quite stout, *versus* weakly serrate and narrower in *Bryobia cinereae*. (Fig. 3D, E).

**Table 1.** Lengths of the internal lateral seta *l*’_1_ on femur I and of the coxisternal setae *1b* and *1c* of *Bryobia cinereae* (lengths are given in micrometers).

	Seta *l*’_1_ on femur I	Coxisternal seta *1b*	Coxisternal seta *1c*
Holotype	31	48	18
Paratype 1	29	46	20
Paratype 2	27	45	19
Paratype 3	31	44	20

##### Etymology.

The species designation *belliloci* refers to a village that felt into ruin, named Bell Lloc (meaning beautiful place in Catalan language) that is close to the place where mites were collected.

#### Bryobia cinereae

Taxon classificationAnimaliaProstigmataTetranychidae

Auger & Migeon, 2014

http://zoobank.org/5C0B1E07-FD04-4829-9959-CE6FFE1E5D60

Figure 3E

##### Remarks.

Additional data to the original description of *Bryobia cinereae* are provided. Despite that usually not included in descriptions, they appeared to be useful for the comparison of this species with *Bryobia belliloci*. Drawings of the two coxisternal setae *1b* and *1c* are shown in Fig. 3E. Measures of their lengths and that of the internal lateral seta *l*’_1_ present on femur I in holotype and paratypes are given in Table 1.

#### Bryobia gigas sp. n.

Taxon classificationAnimaliaProstigmataTetranychidae

http://zoobank.org/12B3D6D0-E26A-4625-BA5A-1A6DF4A57BFD

**Figure 6.**
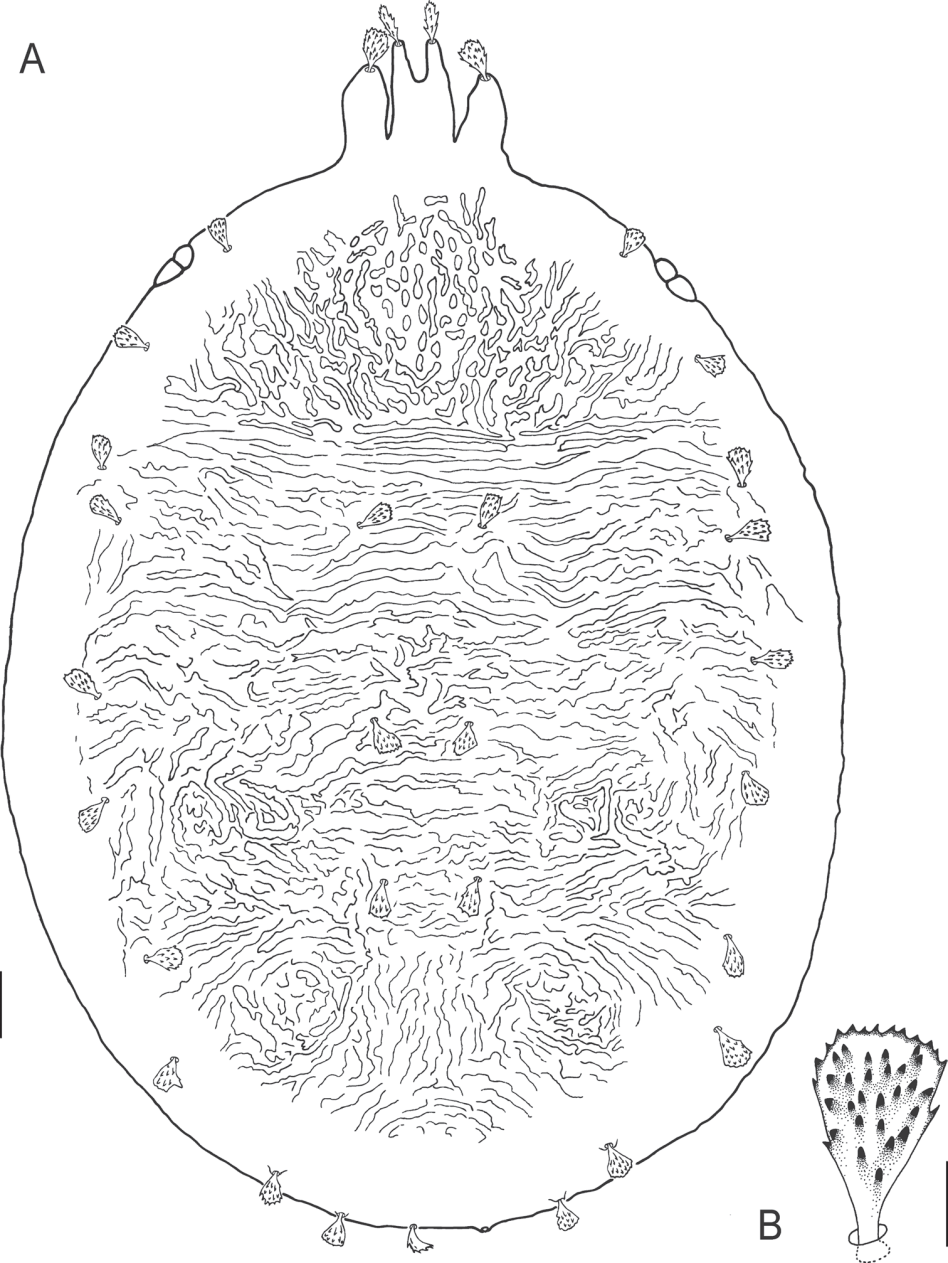
*Bryobia gigas* sp. n., female: **A** dorsal aspect **B** dorsal *c*_2_ seta. Scale bars = 50 µm (**A**), 10 µm (**B**).

**Figure 7.**
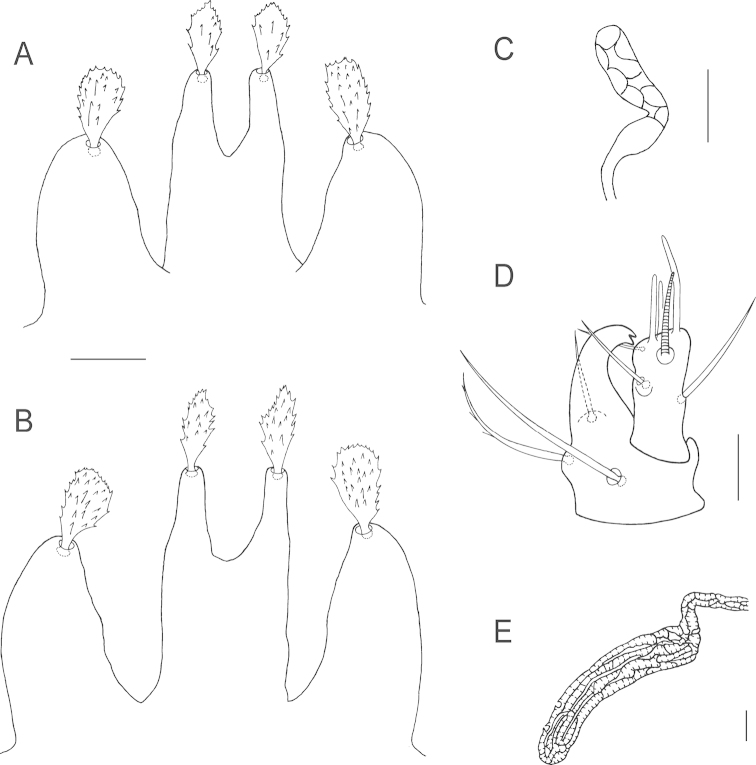
*Bryobia gigas* sp. n., female: **A** prodorsal lobes **B** variation in prodorsal lobes **C** spermatheca **D** palpal tibia and tarsus **E** peritremal distal enlargement. Scale bars = 25 µm (**A–B**), 10 µm (**C–E**).

**Figure 8.**
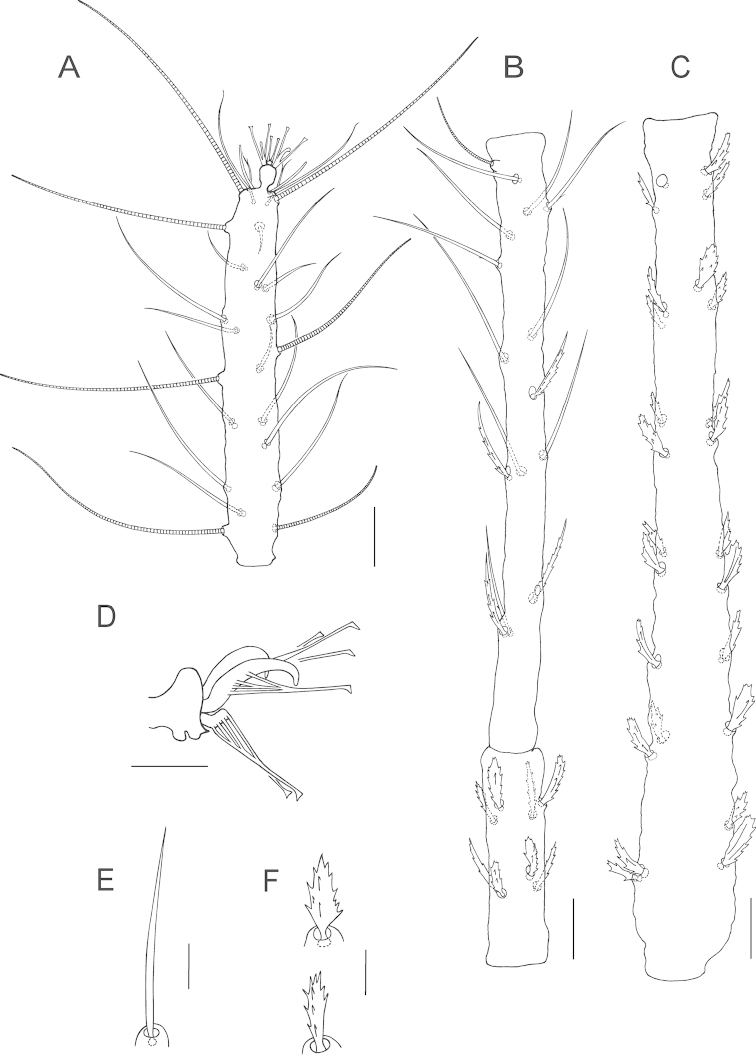
*Bryobia gigas* sp. n., female: **A** tarsus I **B** tibia and genu I **C** femur I **D** claws and empodium I **E** coxisternal seta *1b* **F** coxisternal seta *1c* (variations). Scale bars = 25 µm (**A–C**), 10 µm (**D–F**).

**Figure 9.**
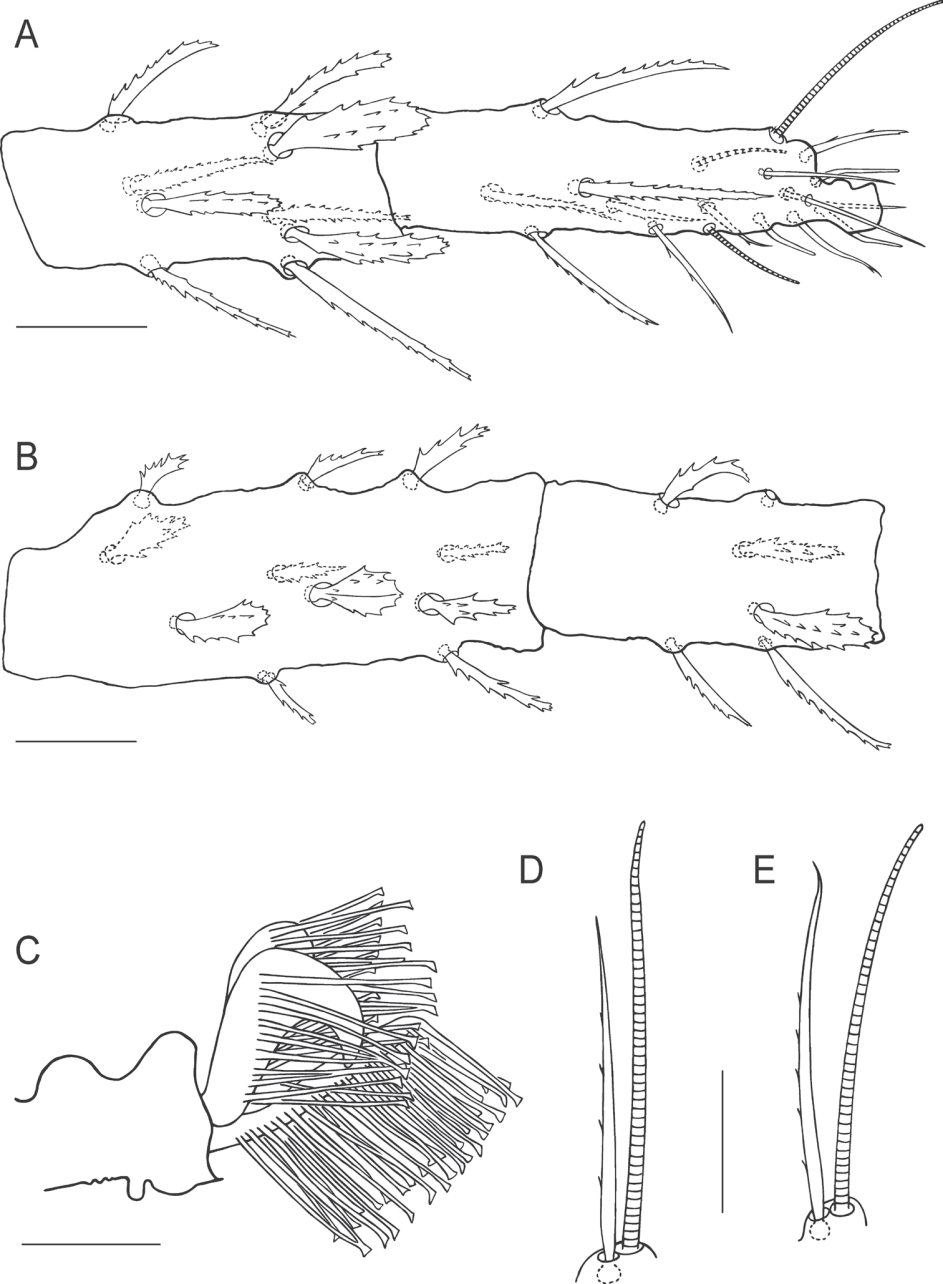
*Bryobia gigas* sp. n., female: **A** tarsus and tibia II **B** genu and femur II **C** claws and empodia II–IV **D** duplex setae on tarsus III **E** duplex setae on tarsus IV. Scale bars = 25 µm (**A–B**), 10 µm (**C–E**).

**Figure 10.**
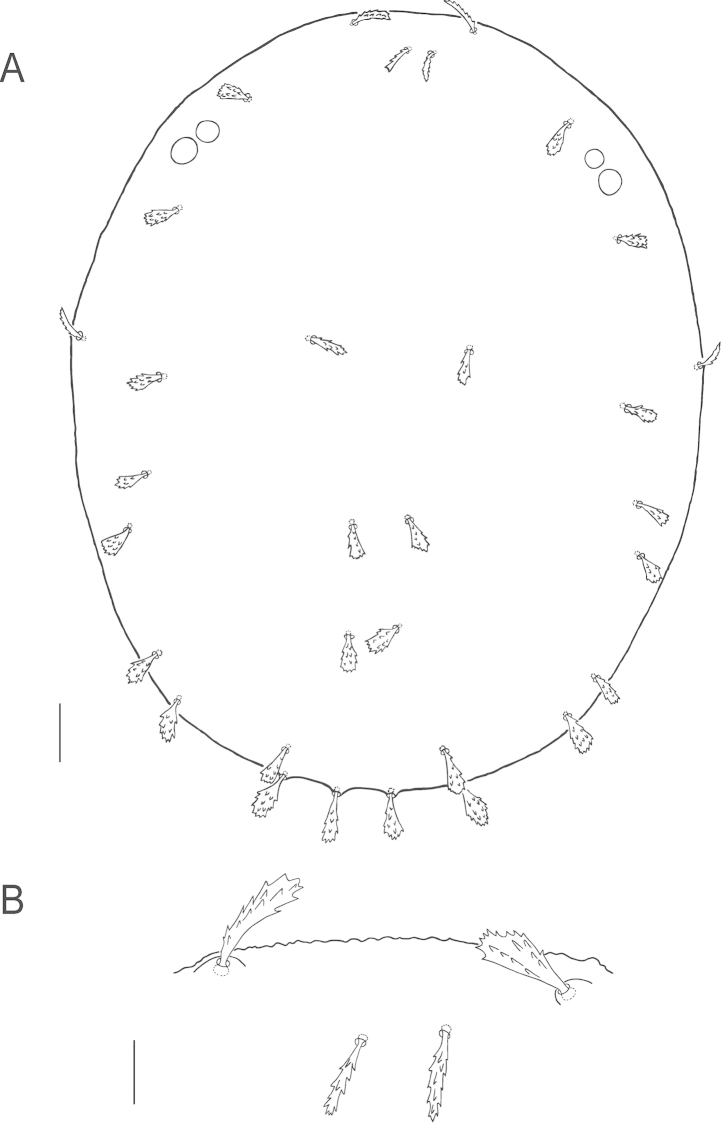
*Bryobia gigas* sp. n., larva: **A** dorsal aspect **B** prodorsal setae (*v*_1,_ *v*_2_). Scale bars = 25 µm (**A**), 10 µm (**B**).

Figures 6 , 7 , 8 , 9 , 10

##### Type material.

Holotype (female), 9 female and 2 larvae paratypes on 12 microscopic preparations from *Bituminaria bituminosa* (L.) C.H. Stirt. (Leguminosae), Four de la caux (43°35.2241N, 3°44.9143E, alt. 90 m), Pignan, Hérault (34), France, 23.XII.2012, leg. P. Auger. All the material deposited in the collection of the CBGP, coll. Auger-Migeon N°1827 for holotype, 1828–1838 for paratypes.

##### Diagnosis.

Body and leg I large, anterior dorsal propodosomal projections over gnathosoma well developed, inner lobes with fused base, candle like-shaped distally, incision between inner vertical setae (*v*_1_) wide, bottom rounded. Dorsal body setae short, spatulate, serrate, inserted on small bulges, subequal in length on hysterosoma, *f*_1_ and *f*_2_ setae marginal not contiguous. Empodia I with a pair of tenent hairs, others with two rows of tenent hairs.

##### Description.

FEMALE. Holotype 880 long (including gnathosoma), width 593. 7 paratypes measured, 860–916 long, width 574–628.

*Dorsum*. Prodorsum with four pairs of setae, anterior propodosomal lobes well developed (Figs 6A, 7A–B). Lobes with basal width about 125 (122–134), outer propodosomal lobes 66 (61–74) high (excluding setae), teat-like shaped, extending about three quarters of inner lobes; inner lobes longer than broad, 73 (63–76) high, 51 (49–56) wide, with fused base about half their length, well separated by deep, wide and bottom rounded incision 27 (27–33) in depth (measured from the bottom of the incision between the inner lobes to their tip, excluding setae). Incision between median and outer lobes deep and narrow. The imaginary transverse line passing to the top of the outer lobes crosses inner near or just above the bottom of the incision. The line joining tips of second pair of propodosomal stae (*v*_2_) located on the outer lobes generally passes just above the bases of the first pair (*v*_1_). Distance between *v*_1_ and *v*_2_ setae insertions 29 (19–29) and 86 (86–103), respectively, *v*_1_ and *v*_2_ setae subequal in length, *v*_1_ and *v*_2_ subspatulate to spatulate, *v*_2_ wider. Dorsal body setae spatulate, palmate, rough, serrate, inserted on small bulge-like structures, subequal in length, *sc*_1_ the shortest (Fig. 6A–B). Dorsocentral setae (*c*_1_, *d*_1_ and *e*_1_) shorter than distances between consecutive setae (length of holotype and variations of 7 paratypes): *v*_1_ 28 (24–30); *v*_2_ 30 (29–31); *sc*_1_ 24 (21–24); *sc*_2_ 24 (20–26); *c*_1_ 28 (24–29); *c*_2_ 29 (22–30); *c*_3_ 24 (21–27); *d*_1_ 26 (23–29); *d*_2_ 28 (22–31); *d*_3_ 26 (22–30); *e*_1_ 29 (25–30); *e*_2_ 27 (25–28); *e*_3_ 28 (23–30); *f*_1_ 27 (23–27); *f*_2_ 27 (23–28); *h*_1_ 26 (23–26). Distances between setae: *c*_1_-*c*_1_ 88 (83–98), *d*_1_-*d*_1_ 67 (67–79), *e*_1_-*e*_1_ 68 (63–74), *c*_1_-*d*_1_ 145 (125–149), *d*_1_-*e*_1_ 111 (11–127). Sacral setae (*f*_1_ and *f*_2_) in marginal position, not contiguous. Dorsal integument on propodosoma with irregularly rounded reticulated granulated pattern medially more elongated laterally and oblique. Folds on hysterosoma mostly transverse, irregularly arched in the caudal part. Two pairs of more or less oval-shaped shallow dimples present between *d*_1_-*d*_3_, and *e*_1_-*e*_3_ setae and one present posteriorly.

*Gnathosoma*. Stylophore longer than wide. Tibial claw of palpus bidentate. Palptarsus subequal in length to tibial claw, about 28 (27–29) long with three tactile setae, three eupathidia and one solenidion (Fig. 7D). Eupathidia *ul*’ζ, *ul*’’ζ shorter than solenidion, *su*ζ longer than solenidion. Peritreme anastomosed distally in a relatively long and slender enlargement, 62 (52–71) long (Fig. 7E).

*Venter*. Striation transverse between 1^st^ (*1a*) and 2^nd^ (*3a*) pairs of setae, between 2^nd^ and 3^rd^ (*4a*) irregular longitudinal striation medially more or less oblique or arched laterally, transverse above and between *4a* and the area anterior to aggenital (*ag*) setae, longitudinal between members of *ag* setae, area immediately anterior to genital flap with irregular longitudinal striation. Sacculus of spermatheca elongated, length 22.5, width 5.5 (Fig. 7C). Three pairs of pseudanal setae (*ps*_1–3_) and two pairs of ventrocaudal (*h*_2–3_) setae present.

*Legs*. Leg I subequal in length to body length, other legs inferior to body length. Leg I 926 (825–947) long (measured from trochanter to tarsus), leg II 392 (345–392), leg III 353 (352–373), leg IV 470 (412–470). Length of segments of leg I as follows: trochanter 50 (41–69), femur 360 (300–360), genu 90 (84–102), tibia 260 (227–274), tarsus 168 (155–180). Leg setal counts as follows (Figs 8A–C, 9A–B):

I 2 − 1 – 25[23–24] − 8[7] – 15[16] + (1) – 20[19] + (5)[(6)] + 2 duplexes;

II 1 − 1 – 11[10] – 6[5] – 9[8] – 15 + (2) + 1 duplex;

III 1 − 1 – 5[4] – 6[5–7] − 9[8] – 13 + 1 duplex;

IV 1 − 1 − 5 – 6[5] − 9[8] – 13 + 1 duplex.

True claws uncinate, claw and empodium I with one pair of tenent hairs, other claws with several pairs of tenent hairs, other empodial pads each provided with two rows of tenent hairs (Figs 8D, 9C). Coxisternal seta *1b* slender 41 (40–50), coxisternal seta *1c* shorter 17 (17–20), serrate, stout (Fig. 8E–F). Tarsi III and IV associated setae serrate and approximate with solenidion forming duplex, the tactile member shorter (about ¾ the length of solenidion) and proximal (Fig. 9D–E).

LARVAE: two larvae measured, 366–370 long (including gnathosoma), width 246–255.

*Dorsum*. Prodorsal lobes absent (Fig. 10A–B). Prodorsal setae serrate, subspatulate except *v*_1_ short, elongated. *v*_1_ setae inserted without tubercle, *v*_2_ inserted on small bulges. Other dorsal setae inserted on tiny bulges more obvious posteriorly. Hysterosomal setae serrate, wider caudally, subspatulate to spatulate, *e*_3_ and *f*_1_ wider, *f*_2_ and *h*_1_ longer. Setae *f*_1_ in normal position.

Lengths of dorsal setae: *v*_1_ 13; *v*_2_ 18–20; *sc*_1_ 16–18; *sc*_2_ 15; *c*_1_ 15–16; *c*_2_ 16; *c*_3_ 16; *d*_1_ 19–17; *d*_2_ 16–17; *d*_3_ 15–16; *e*_1_ 18–21; *e*_2_ 16–18; *e*_3_ 19; *f*_1_ 18–21; *f*_2_ 22–23; *h*_1_ 22–23.

*Legs*. Length inferior to body length, leg I 235–248 long. Leg setal counts as follows:

I 1 − 0 − 3 − 4 − 5 + (1) – 7 + 1 duplex;

II 0 − 0 – 3 − 4 − 5 – 7 + 1 duplex;

III 0 − 0 – 2 – 2 − 5 – 6.

True claws uncinate with one pair of tenent hairs, empodia with two rows of tenent hairs. On femur I, internal seta smooth, dorsal seta serrate.

##### Remarks.

*Bryobia gigas* is morphologically close to three species, that belong to a species-group characterized by: 1) prodorsal inner and outer lobes very well developed, outer teat like not triangular, anteromedian well separated; 2) associated setae forming a duplex on tarsus IV, tactile member shorter than solenidion; 3) similar setal counts especially on leg I with 29 and 16 setae on tarsus and tibia, respectively, and on leg II with 19 and 9 setae on tarsus and tibia, respectively; 4) a pair of tenent hairs on the empodium of the foreleg and two rows of tenent hairs on the other empodia. These species are *Bryobia osterloffi* Reck, 1947, *Bryobia vasiljevi* Reck, 1953 and *Bryobia lagodechiana* Reck, 1953.

Among this species-group *Bryobia gigas* and *Bryobia lagodechiana* have a similar large body size. *Bryobia gigas* is mainly distinctive from *Bryobia lagodechiana* by the shape of the inner incision between the anteromedian prodorsal lobes which is wide and bottom rounded in the former whereas narrow in the latter. In addition, the line that passes to the tips of *v*_2_ setae does not reach the bases of *v*_1_ setae in *Bryobia lagodechiana*. They also have a different setal count on genu I with 7–8 and 4–5 setae present in *Bryobia gigas* and *Bryobia lagodechiana*, respectively.

*Bryobia gigas* can be easily distinguished from *Bryobia osterloffi* and *Bryobia vasiljevi* by its body and leg sizes which are far smaller in the latters. Moreover, in *Bryobia osterloffi*, the incision between the inner prodorsal lobes is wide but less deep than in *Bryobia gigas* (the line that passes to the top of the outer lobes does not reach the bottom of the incision between inner lobes), it is not bottom-rounded but with a flat bottom and the distal part of the peritreme is less elongated. *Bryobia gigas* also differs from *Bryobia vasiljevi* by the incision between the inner prodorsal lobes which is narrow. According to Livshits and Mitrofanov (1971), 6 setae are present on the femora III and IV of *Bryobia vasiljevi* whereas 5 are present in *Bryobia gigas*. However, type’s examination of *Bryobia vasiljevi* in Reck’s collection gave conflicting data because 5 setae only are present on femora III and IV of the 21 type specimens. As a consequence, this morphological criterion cannot be used to distinguish between the two species.

##### Etymology.

The specific epithet *gigas*, name given to “Giants” in Greek mythology, refers to the quite unusual large body and legs sizes of this species.

### Key to species of the French *Bryobia* (♀):

**Table:** 

1	Prodorsal lobes poorly developed or absent	**2**
-	Prodorsal lobes well developed over gnathosoma	**4**
2	Dorso-hysterosomal setae elongate, short, variable in length, *h* 1 the largest, *c*_1_ and *d*_1_ shorter than half the distance between consecutive setae	***Bryobia sarothamni* Geisjke, 1939**
-	Dorso-hysterosomal setae elongate, long, subequal in length, *c*_1_ and *d*_1_ longer than half the distance between consecutive setae	**3**
3	Dorsocentral setae serrate, stout, shorter than distance between consecutive setae	***Bryobia cinereae* Auger & Migeon, 2014**
-	Dorsocentral setae serrate, narrow, *d*_1_ surpass bases of *e*_1_ setae	***Bryobia belliloci* sp. n.**
4	Femur I with 4 long setae present on its interior dorsal row	**5**
-	Femur I without this character	**11**
5	Empodium I with one pair of tenent hairs	**6**
-	Empodium I with more than one pair of tenent hairs	**7**
6	Propodosomal inner lobes mammelliform, inflated, largely fused; deutonymphal dorsohysterosomal setae *e*_3_ and *f*_1_ subspatulate, 5 tactile setae and 1 solenidion on tibia I	***Bryobia provincialis* Eyndoven & Vacante, 1985**
-	Propodosomal inner lobes not mammelliform, well separated distally; deutonymph with setae *e*_3_ and *f*_1_ elongate, narrow, 9 tactile setae and 1 solenidion on tibia I	***Bryobia mercantourensis* Auger & Migeon, 2014**
7	Outer propodosomal lobes triangular	**8**
-	Outer propodosomal lobes not triangular, broad or mammelliform	**10**
8	Female body length superior to 630 µm, spermatheca subglobular	***Bryobia berlesei* Eyndhoven, 1957**
-	Female body length inferior to 600 µm, sacculus of spermatheca elongate	**9**
9	Sacculus of spermatheca elongate short, 14–22 µm long, male unknown	***Bryobia pandayi* Eyndoven & Vacante, 1985**
-	Sacculus of spermatheca elongate long, 30–40 µm long, male present	***Bryobia pyrenaica* Eyndoven & Vacante, 1985**
10	Female body length inferior to 510 µm, spermathecal sacculus pyriform, distal part of peritreme about 50 µm long	***Bryobia dekocki* Eyndoven & Vacante, 1985**
-	Female body length superior to 570 µm, spermathecal sacculus elongate, peritremal anastomosis 60–64 µm long	***Bryobia ulicis* Eyndoven, 1959**
11	Tarsus IV associated seta well separated from solenidion, distal	***Bryobia rubrioculus* (Scheuten, 1957)**
-	Tarsus IV associated seta approximate with solenidion forming duplex	**12**
12	Outer prodorsal lobes well developed, mammelliform, separate from anteromedian lobes by a deep incision	**13**
-	Outer prodorsal lobes triangular	**14**
13	Incision between anteromedian lobes wide and bottom-rounded, body and leg I large (860–916 and 825–947 µm long, respectively)	***Bryobia gigas* sp. n.**
-	Incision between anteromedian lobes narrow, body and leg I smaller (690–840 and 760–778 µm long, respectively)	***Bryobia vasiljevi* Reck, 1953**
14	Female body and leg I about 900 µm long or more, male present	***Bryobia graminum* (Schrank, 1781)**
-	Female body and leg I inferior to 750 µm long	**15**
15	Larval dorsal setae narrow, needle-like, narrowly subspatulate on protonymphs, on *Ribes* sp.	***Bryobia ribis* Thomas, 1896**
-	Larval dorsal setae narrowly subspatulate, spatulate and wider distally on protonymphs, on *Hedera helix*	***Bryobia kissophila* Eyndhoven, 1955**

## Supplementary Material

XML Treatment for Bryobia belliloci

XML Treatment for Bryobia cinereae

XML Treatment for Bryobia gigas
